# On the Development and Anatomy of the Prostate Gland

**Published:** 1905-09

**Authors:** 


**On the Development and Anatomy of the Prostate Gland.** By W. G. Richardson, M.B., B.S. Pp. xii., 121. London: J. & A. Churchill. 1904.

The author was awarded the Heath Scholarship by the Council of the Durham College of Medicine for this essay, which deals not only with the anatomy and development of the prostate, but also with its diseases and their surgical treatment.

The anatomy, comparative anatomy, and development of the gland are minutely described and amply illustrated. The illustrations, derived in nearly all cases from the author's original drawings, are excellent. We had an opportunity of seeing many of the original drawings and the specimens from which they were made at the Oxford meeting of the British Medical Association in 1904, and we much admired them. Those showing the anatomy of the internal organs of generation of various animals were specially interesting and instructive.

In considering the causes of senile enlargement of the prostate, the writer dismisses as improbable most of the causes which have been hitherto suggested, including previous gonorrhœa. He believes that adenomatous changes in the gland are a normal sequence of declining years, and that hypertrophy is an excessive change of similar kind.

In view of recent discussions on the question as to the completeness with which the prostate is removed in the operation of supra-pubic prostatectomy, it is interesting to find that the author believes it is not possible to completely remove the gland without damaging its fascial sheath containing the large veins of the prostatic plexus. A case is cited and a specimen figured which support the contention that the operation called total extirpation is not in reality what it is supposed to be, and that a bed of prostatic tissue is always left behind when the gland is removed.

We may pronounce the work as a whole to be very readable and instructive.

